# A *SIX1* homolog in *Fusarium oxysporum* f.sp. *cubense* tropical race 4 contributes to virulence towards Cavendish banana

**DOI:** 10.1371/journal.pone.0205896

**Published:** 2018-10-22

**Authors:** S. Widinugraheni, J. Niño-Sánchez, H. C. van der Does, P. van Dam, F. A. García-Bastidas, S. Subandiyah, H. J. G. Meijer, H. C. Kistler, G. H. J. Kema, M. Rep

**Affiliations:** 1 Molecular Plant Pathology, Swammerdam Institute for Life Sciences, University of Amsterdam, Amsterdam, the Netherlands; 2 Faculty of Agriculture, Nusa Cendana University, Kupang, Indonesia; 3 ARS-USDA Cereal Disease Laboratory, University of Minnesota, St. Paul, Minnesota, United States of America; 4 Wageningen University and Research, Wageningen Plant Research, Wageningen, the Netherlands; 5 Dept. Plant Protection, Fac. Agriculture, Gadjah Mada University, Yogyakarta, Indonesia; 6 Research Center for Biotechnology, Gadjah Mada University, Yogyakarta, Indonesia; Oregon State University, UNITED STATES

## Abstract

The fungus *Fusarium oxysporum* f.sp. *cubense* (Focub) causes Fusarium wilt of banana. Focub strains are divided into races according to their host specificity, but which virulence factors underlie these interactions is currently unknown. In the *F*. *oxysporum* f.sp. *lycopersici* (Fol)-tomato system, small secreted fungal proteins, called Six proteins, were identified in the xylem sap of infected plants. The Fol Six1 protein contributes to virulence and has an avirulence function by activating the I-3 immune receptor of tomato. The Focub tropical race 4 (TR4) genome harbors three *SIX1* homologs: *SIX1a*, *b* and *c*. In this study, the role of Focub*-SIX1a* in pathogenicity was evaluated since this homolog is present in not only TR4 but also in other races. A deletion mutant of the *SIX1a* gene from Focub TR4 strain II5 was generated (Focub*ΔSIX1a*) and tested *in planta*. Mutants were found to be severely compromised in their virulence. Ectopic integration of the Focub*-SIX1a* gene in the Focub*ΔSIX1a* strain restored virulence to wild type levels. We conclude that Focub-*SIX1a* is required for full virulence of Focub TR4 towards Cavendish banana.

## Introduction

*Fusarium oxysporum* f.sp. *cubense* (Focub) is a major fungal threat to banana cultivation. Focub comprises four physiological races based on the banana cultivars that can be infected. Focub race 1 affects banana triploid *Musa* AAA (sub-group Gros Michel) and *Musa* AAB (sub-group Pome)[1,2]; race 2 affects cooking banana triploid *Musa* ABB (sub-group Bluggoe) and *Musa* ABB (sub-group Saba) [1,2]; race 3 affects *Heliconia* species, an ornamental plant, and is therefore no longer considered as *cubense* [3,4]; and race 4 affects the above-mentioned varieties as well as in the Cavendish sub-group (*Musa* AAA sub-group Cavendish). Focub tropical race 4 (TR4) is economically destructive since it can infect the globally grown Cavendish cultivars (Grand Nain, Williams, and Valery). This sub-group is resistant to race 1 and race 2 and was therefore introduced to replace the race 1-sensitive Gros Michel cultivars [5]. Focub TR4 emerged for the first time in Taiwan and then spread over South East Asia [5]. It has now been detected as well outside South East Asia *i*.*e* in Pakistan, Jordan, Oman, Lebanon, Mozambique, in the Northern Territory in Australia, and more recently in the Tully region in Queensland, Australia [6–8].

Fundamental understanding of plant-microbe interactions requires the identification of proteins that are required for virulence [9]. Important questions on how the host-pathogen interaction works in this pathosystem are currently unanswered. Studies aiming at the identification of virulence genes in Focub are as yet limited [10,11]. Lessons can be learnt however, from studies on other *formae speciales* in the *F*. *oxysporum* complex.

In *F*. *oxysporum* f.sp. *lycopersici* (Fol) 14 “Secreted in xylem” (*SIX*) genes were identified, encoding small *in planta* secreted proteins, also called ‘effectors’. For some of these, a distinct role in virulence and/or avirulence in the Fol-tomato pathosystem has been demonstrated [12–19]. Most *SIX* genes of Fol reside on a single ‘pathogenicity’ chromosome, chromosome 14 in strain Fol4287 [20]. Among them is *SIX1*, encoding a protein that contributes to virulence of Fol [15,21]. Six1 was found to trigger disease resistance in tomato plants that carry the *I-3* resistance gene [22,23]. Thus, Six1 also functions as avirulence factor, and is alternatively designated as Avr3 [12,24]. Homologs of *SIX1* are present in many *formae speciales* (ff.spp) of *F*. *oxysporum* including Focub, *F*. *oxysporum* f.sp *pisi* (Fop), Fol, *F*. *oxysporum* f.sp. *conglutinans* (Focon) and *F*. *oxysporum* f.sp. *melonis* (Fom) [25]. Deletion and complementation of *SIX1* in Focon showed that Six1 contributes to virulence against cabbage [26]. In addition to *SIX1*, strains of Focub possess homologs of *SIX2*, *SIX6*, *SIX7*, *SIX8*, *SIX13* [10,12,26] and a recent study by Czislowski *et al*. [27], revealed the presence of *SIX4*, *SIX9* or *SIX10* homologs in certain Focub strains.

The Focub race 4 strain B2 (TR4) has three homologs of *SIX1*: *SIX1a*, *b* and *c*, while race 1 isolates only possess *SIX1a* [11]. *SIX1c* (FOC4_g10000575) is highly expressed *in planta* 48 hours after inoculation with a Focub TR4 strain, whereas *SIX1b* (FOC4_g10000324) is not [11]. *SIX1a* (FOC4_g10000240) is expressed both in race 1 and race 4 strains during compatible interaction with susceptible banana plants [11]. Because *SIX1a* is present in all races of Focub and was found to be expressed during banana infection, we investigated the role of *SIX1a* in the Focub TR4-Cavendish interaction.

## Materials and methods

### Fungal strains and growing conditions

Wild type Focub TR4 strain II5 (NRRL#54006), isolated from *Musa spp* in Indonesia was used in this study. This isolate is designated as VCG 01213 and is reported as TR4 (Broad Institute,[20,28]). The Focub*∆SIX1a* mutant and its complementation strains were derived from this isolate, and both the wild type and mutants were grown on Czapek Dox Agar (CDA) plates for temporary storage. For long-term storage, spores grown in NO_3_ medium (0.17% yeast nitrogen base without ammonia and amino acids, 3% sucrose, 100 mM KNO_3_) were collected by filtering the culture through sterile miracloth and then mixed with 25% glycerol (1:1) for storage at -80°C freezer.

### Banana cultivar nursery

*In vitro* Grand Nain banana plantlets (*Musa* AAA, sub-group Cavendish) were obtained from Rahan Meristem (1998) Ltd, Israel. Plantlets were cleansed from agar by rinsing in water supplied with 2.5% sodium hypochlorite disinfectant. Subsequently, plants were potted in soil (Swedish sphagnum peat 5%, grinding clay granules 41%, garden peat 5%, beam structure 4%, steamed compost 33%, PG-Mix-15-10-20 12%) and acclimatized in the greenhouse with the required condition 28 ±2°C, 16h light and ~85% relative humidity regime; respectively. Plants were cultivated for ~2.5 months until at least the first five leaves were completely open before they were used in a pathogenicity assay. Within this time watering was done each day and fertilizing was applied weekly.

### Identification of *SIX1* homologs in the Fo genomes

The whole genome sequence of several strains possessing a *SIX1* homolog were retrieved from Genbank (accession numbers are listed in Table 1). The gene sequences were collected from each accession using BLASTN, aligned using ClustalO v1.2.1 [29] and phylogeny was inferred using PhyML v20120412 [30]. Visualisation was done with ETE v3 [31]. To verify the presence of *SIX1* homologs in *F*. *oxysporum* strains that belong to various *formae speciales* we analysed the genomes of the strains listed in Table 1.

**Table 1:** Accession numbers of genome assemblies used in this study (see also S2 Table).

Strain	*Forma specialis*	Genbank accession
Fol4287	*lycopersici*	GCA_000149955.2
Fop HDV247	*pisi*	GCA_000260075.2
Fo5176	*conglutinans*	GCA_000222805.1
Fom001 (NRRL26406)	*melonis*	GCA_000260495.2
Fom010	*melonis*	MALD01000000
Fomom001	*momordicae*	NJCB01000000
Folag001	*lagenariae*	NJCJ01000000
Fomel001	*melongenae*	NJCC01000000
Fophy KOD886	*physali*	NJBW01000000
Focub II5	*cubense* TR4	GCA_000260195.2
Focub B2	*cubense* race 4	GCA_000350365.1
Focub N2	*cubense* race 1	GCA_000350345.1

### Creation of a *SIX1a* deletion mutant

A *SIX1a* (FOIG_16557) knock-out strain was generated by transforming TR4 strain Focub II5 (VCG 01213) with the construct pKOSIX1a.OSCAR. This construct was built using the Gateway cloning system [32], and contains an upstream flank of 470 bp generated with primers SIX1attB3 and SIX1attB4, a hygromycin (HPH) resistance cassette consisting of P_*GpD*_*-HPH-T*_*TrpC*_ locus [33], and a down-stream flank of 558 bp generated with primers SIX1attB1 and SIX1attB2, (see primer list in S1 Table). *Agrobacterium tumefaciens* mediated transformation (ATMT) was used to deliver the construct into the Focub II5 [33–35]. Transformants surviving on hygromycin-supplemented medium were monospored and assessed for homologous recombination using primer pairs located at the flanking region and in the hygromycin cassette (S1 Table) *In-locus* transformants were further confirmed by the loss of the *SIX1a* Open Reading Frame (ORF) and the presence of the hygromycin resistance cassette (S1 Table).

### Complementation of the *SIX1* deletion

A complementation construct was generated in the pRW1p vector [36]. The Focub-*SIX1a* locus (812 bp upstream—*SIX1a* ORF—589 bp downstream) was amplified from gDNA of strain Focub II5 using primer pairs FP#6522 and FP#6523 (S1 Table) generating a 2209 bp product that was inserted into the vector between the *PacI* and *EcoRI* sites preceding the Zeocin and Phleomycin (BLE) resistance cassette consisting of P_GpD_-*BLE*-T_TrpC_ [33]. This construct was transformed into the Focub*-SIX1a* knockout mutant through ATMT. Transformants that were resistant to Zeocin were selected and monospored. PCR analysis was performed to check the transformants selected from the Zeocin plates targeting the Focub*-SIX1a* locus, the HPH and the BLE cassettes, respectively (S1 Fig; S1 Table).

### Pathogenicity assay on banana

A double pot system for banana inoculation with Focub as developed by [37] and modified (Garcia et al -submitted) was used in this experiment. This double pot system employs two pots of different size, the smaller one being used for planting and the bigger pot is used for prevention of contamination. The smaller pot was filled with soil, and a saucer-disc was placed on the bottom of the bigger pot.

Focub was grown on CDA plates for 7 days. Spores for inoculation were produced as described in [38] and conidial concentration was adjusted to 10^6^ spores/ml. A second inoculum in the form of corn- kernels inoculated with Focub was prepared by growing a plug of fungal inoculant on a sterile corn-kernel at 25°C in dark for 5 days. Prior to inoculation, the plant roots were wounded to facilitate infection, and the additional corn-kernel fungal inoculum was placed in near the wounded root to enhance the infection.

The first bioassay to test the pathogenicity of Focub*ΔSIX1a* transformant uses four months-old Cavendish banana cv. Grand Nain (AAA) after acclimatisation. In this assay the clean wounded roots of banana plants were dipped into the inoculum with required concentration of 10^6^ spores/ml for 10–30 minutes in a tray, and then the inoculated plants were placed in the pot containing standard soil mix from Unifarm, WUR (Swedish sphagnum peat 5%, grinding clay granules 41%, garden peat 5%, beam structure 4%, steamed compost 33%, PG-Mix-15-10-20 12%). In the second bioassay examining the Focub II5 complementation strains, two months-old of banana of the same cultivar was employed. Here we used inoculum pouring instead of dipping to inoculate the plant following the method used by Garcia et al-(submitted). One hundred ml of liquid culture containing fungal inoculum at a concentration 10^6^ spores/ml was poured onto the root system. Both pathogenicity assays were done in controlled conditions, under a 28 ±2°C, 16h light and ~85% relative humidity regime in a PKM III green-house.

External symptoms such as chlorosis and yellowing were observed normally starting two-three weeks after inoculation. Plants were harvested at the minimum 9 weeks after inoculation, as the plants inoculated with the wild-type Focub II5 were showing wilting symptom. Internal scoring measures the discoloured corm area of the individual plants at the end of observation. The scoring system applies both to the external and to the internal symptoms in which scale 0–4 represents the degree of severity (See S2 Fig)

## Results

### *SIX1* homologs in Focub

**Fig 1:**
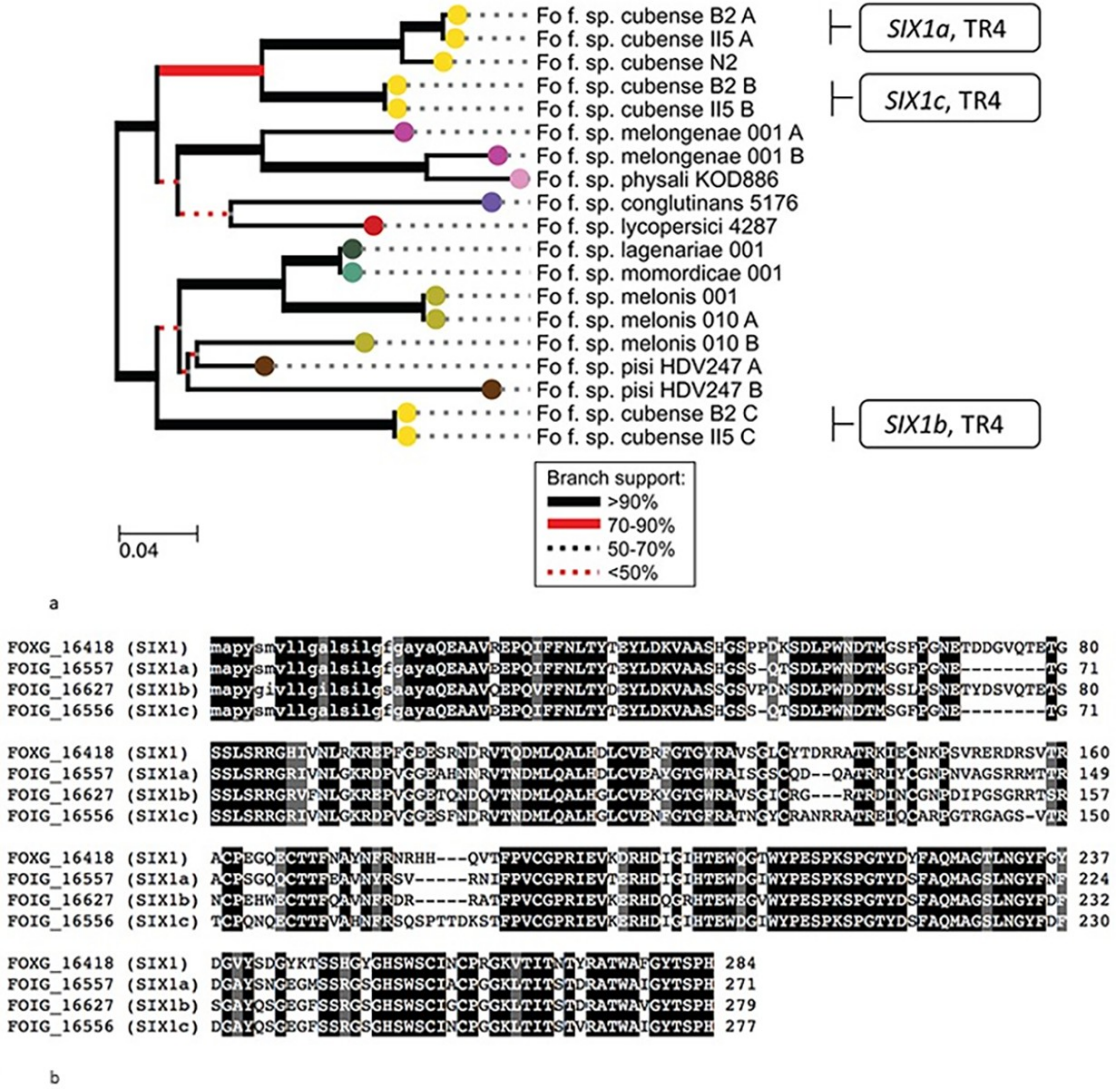
Phylogenetic relationships of *SIX1* homologs in twelve strains of *F*. *oxysporum*. (A) For each sequence type of *SIX1*, one representative genome was used to extract the gene sequence from. These sequences were aligned with ClustalO (total length: 882 nucleotides, including gaps), phylogeny was inferred with PhyML using 100 bootstraps and the tree was visualized with ETE v3. The thicker lines are shown in cases where the tree actually branches. The scale bar relates to the number of nucleotide changes per site in the alignment. (B) Amino acid sequences of Focub Six1 homologs in comparison to Fol Six1. Focub Six1a, Six1b and Six1c of TR4 have 74%, 63% and 73% sequence similarity, respectively, to Fol Six1. Predicted signal peptides are in lower case.

In accordance with earlier findings, the Focub II5 genome carries three homologs of Fol-*SIX1*, annotated as Focub*-SIX1a/b/c*, with nucleotide identity to Fol *SIX1* of 86%, 81% and 84%, and amino acid identity to Fol Six1 of 74%, 63% and 73% respectively (Fig 1). Our phylogenetic analysis clustered Focub-*SIX1a* and Focub-*SIX1c* with *SIX1* of *F*. *oxysporum* f.sp *lycopersici* and *SIX1* of *Fo*. f.sp. *conglutinans* strain 5176. The Focub-*SIX1a* nucleotide sequence is very similar in strains II5 (FOIG_16557) and B2 (FOC4_g10000240) -both are TR4 isolates- with only one nucleotide difference, that leads to an S/A amino acid shift (position 93). Furthermore, TR4 Focub*-SIX1a* and Focub*-SIX1c* are in a separate cluster from Focub-*SIX1b*. The Focub*-SIX1a* sequence of TR4 is only slightly different from the *SIX1* ortholog of Focub race 1 isolate N2.

### Disruption of *SIX1a* results in reduced virulence

Our attempts to delete *SIX1a* in the TR4 isolate Focub II5 resulted in one transformant showing *in locus* integration of the hygromycin-resistant cassette (*HPH*) at the *SIX1a* locus and consequent deletion of the *SIX1a* ORF, out of one hundred hygromycin resistant transformants analysed (S1 Fig). We used this strain, called Focub*ΔSIX1a*, to test whether *SIX1a* is required for virulence of TR4 in a bioassay on Cavendish banana.

**Fig 2:**
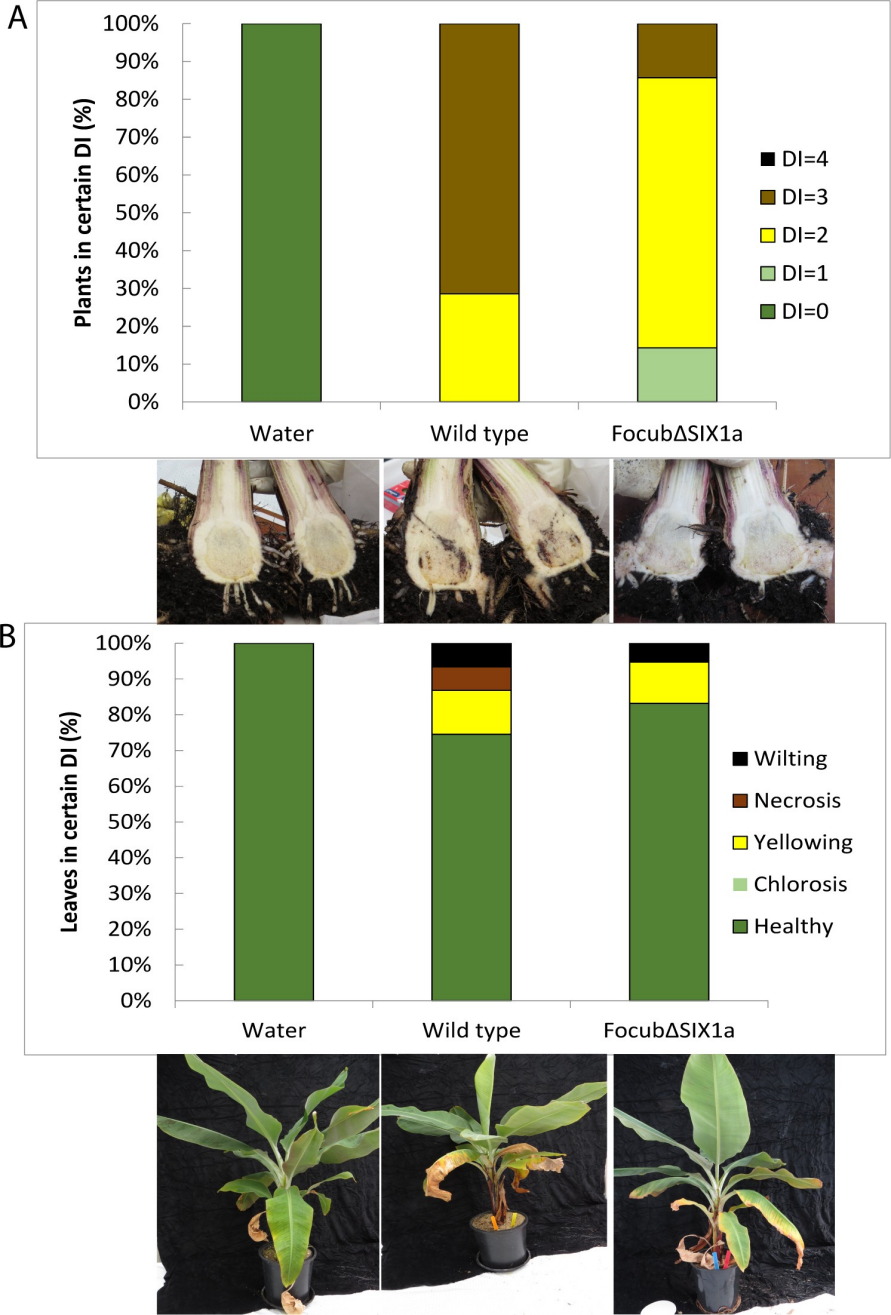
FocubΔ*SIX1a* is less virulent than the wild type strain towards Cavendish banana. Four-months old banana plants were inoculated with Focub TR4 wild type strain II5 or Focub*∆SIX1a*. The percentage of plants in each disease index was scored. Disease index of the internal tissue was determined by scoring of the corm browning area shown in (2a). External disease score, shown in (2b), indicates leaf symptoms: chlorosis, yellowing, wilting or necrosis. The Y axis indicates the percentage of symptomatic plants. Six plants were used for each treatment. For each combination an example is shown of the symptoms below the graph.

The Focub*ΔSIX1a* strain and the wild type isolate Focub II5 were applied separately to Cavendish cv. Grand Nain plants acclimatized for two months in the nursery. A scoring system for external and internal symptoms was used (S2 Fig) to determine the Disease Index (DI) in a range of 0 to 4. External symptoms mostly relate to the state of the leaves, from green to necrosis. Internal symptoms relate to the size of the area of discoloration. Weekly observation of external symptoms was done starting at the first symptom appearance on leaves, *i*.*e* chlorosis indicating score 1, up to 9 weeks (Fig 2). No disease symptoms were observed in the water treatment (negative control). In contrast, wild type Focub II5 treated plants showed severe external symptoms (score 4) after 9 weeks. Plants inoculated with the Focub*∆SIX1a* mutant showed less symptoms both in externally and internally (only 1 out of 6 plants showed an internal Disease Index of 1, the rest had an index of 0), whereas upon infection with the wild type 50% of the plants showed a DI ≥2. This result suggests that *SIX1a* is required for full virulence of Focub II5 in banana.

### *SIX1a* complementation restores virulence of the mutant

**Fig 3:**
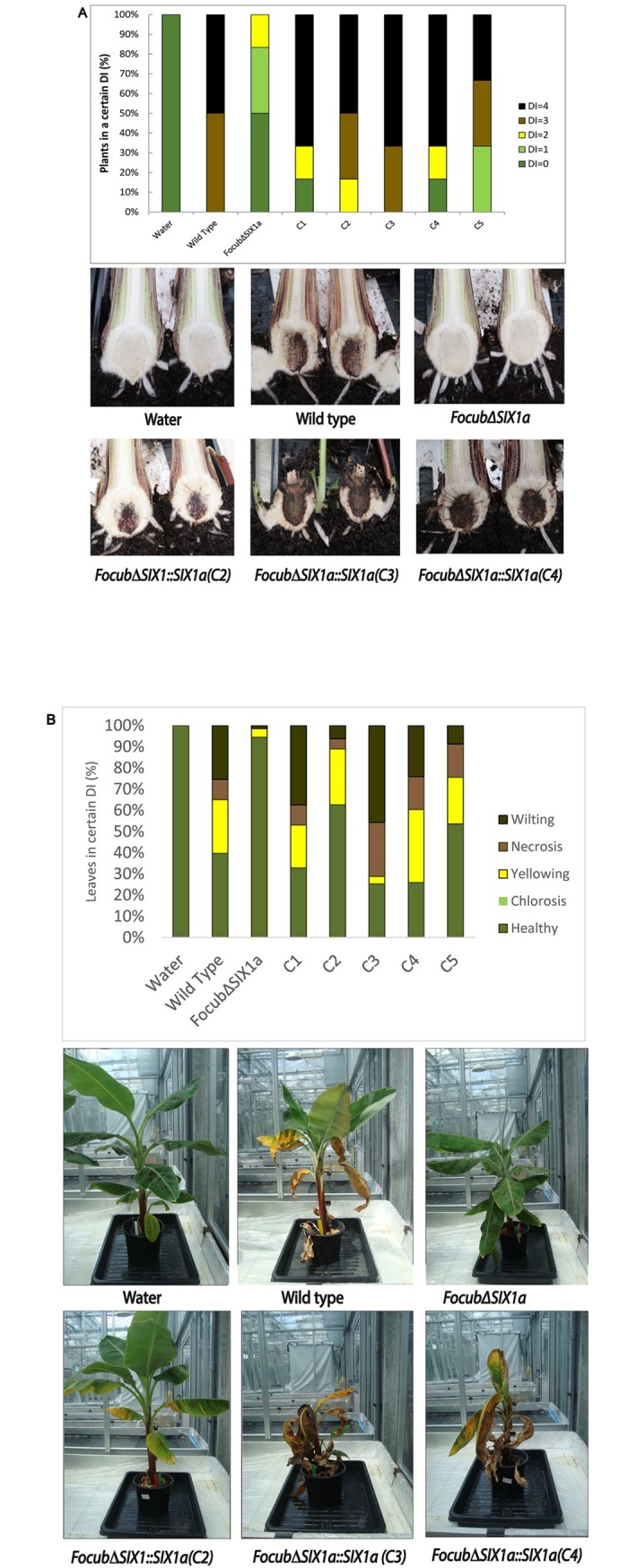
*SIX1a* restores full virulence to *FocubΔSIX1a*. Two-months old banana plants were inoculated with Focub TR4 wild type strain II5, Focub∆SIX1a or complemented strains (C1-C5). The percentage of plants in each disease index was scored for internal (A) and external (B) symptoms as described in Fig 2. The Y axis indicates the percentage of symptomatic plants. Six plants were used for each treatment. Examples are shown below for each treatment.

To validate whether the reduced virulence of the Focub*∆SIX1a* mutant was due to the deletion of *SIX1a*, complementation strains were generated (see Materials and Method; S1 Fig) and a bioassay on Cavendish banana was performed. In total seven independent complementation strains were generated by ectopic integration of *SIX1a* in the genome of Focub∆*SIX1a*, and five were confirmed by PCR to regain the *SIX1a* locus (transformants C1 –C5). These five transformants were tested in a pathogenicity assay. When tested on banana plants, pathogenicity of complemented Focub∆*SIX1a* strains was restored to levels comparable to the wild type Focub II5. In contrast, the Focub∆*SIX1a* again showed clearly reduced virulence (Fig 3, S3 Fig). This establishes *SIX1a* as a pathogenicity factor of Focub TR4.

## Discussion

Fusarium wilt on banana has been reported as a devastating disease in the banana industry mainly by the Tropical Race 4 of Focub. It has been reported that based on the effector profiles this f.sp formed a cluster which separate it from another *Fusarium oxysporum* ff.spp [25]. Focub has also been reported to be polyphyletic and, given the similarity in the *SIX* gene profile, horizontal transfer may have led to its polyphyletic nature instead of convergent evolution [27,39].

We have characterized Focub*-SIX1a*, a homolog of Fol*-SIX1* which has a virulence and an avirulence function during infection of tomato by *F*. *oxysporum* f.sp. *lycopersici* [12]. In this study homologs of Fol-Six1 from twelve Fo isolates were compared; one to three homologs are present per *forma specialis*. In Focub TR4, three homologs are present. While in other lineages *SIX1* homologs are typically identical within a *forma specialis* [25], Focub has three homologs that belong to the different clades in the phylogeny. The presence of the three homologs of *SIX1* in Focub was also shown in earlier reports [11,27]. *SIX1a* is present in all currently known races of Focub, comprising race 1, race 2, sub-tropical race 4 and tropical race 4, in several related sequence types. Czislowski *et al*. [27] also noted the diversity of *SIX1* in Focub and identified nine sequence types in total. So far, their function in pathogenicity remains undetermined.

In tomato, Fol*-SIX1* is upregulated *in planta* when compared to axenic cultures [15]. Also in *F*. *oxysporum* f.sp. *conglutinans* the *Focon-SIX1* expression significantly increased during cabbage infection and Focon-*SIX1* deletion mutants are severely affected in virulence [26]. Similarly, the reduced virulence of Focub*∆SIX1a*, is restored by complementation. These observations, combined with similar results in tomato, onion and cabbage infecting strains [12,26,40], suggest that *SIX1a* has a general virulence function in *F*. *oxysporum*, even though not all *formae speciales* contain a *SIX1* homolog. As for Focub, it remains to be investigated whether *SIX1a* homologs in race 1 and *SIX1b* and *SIX1c* in TR4 are also important for virulence. Another question for further exploration is whether the tomato I-3 immune receptor, which recognizes Six1 (also known as Avr3) from Fol, also recognizes *SIX1* homologs from other *formae speciales* including f.sp. *cubense*. If so, I-3 could be used to combat Fusarium wilt of banana.

## Supporting information

S1 Fig PCR control of Focub*ΔSIX1a* and Focub*ΔSIX1a*::*SIX1a*. In the Focub*ΔSIX1a* strain (KO), the *SIX1a* ORF has been replaced by a hygromycin resistance cassette. The Focub*ΔSIX1a*::*SIX1a* strains (C1-7) have regained the gene by transformation. The upper panel shows the presence of the hygromycin resistance cassette both in the knock-out mutant and in the ectopically transformed strains; the lower panel shows that Focub*ΔSIX1a* has lost the *SIX1a* locus while in the complemented strains it is present. II5 is the wild type strain.
(PDF)

S2 Fig Disease scoring system for external and internal symptoms in banana bioassay. (PDF)

S3 Fig Phenotypic visualization of internal symptoms in the Focub*ΔSIX1a* and *SIX1a* transformants of Focub*ΔSIX1a*. (PDF)

S1 Table List of primers being used in this study. (DOCX)

S2 Table Sequences used in this study. (XLSX)
