# Supplementary figures and images for: A SIX1 homolog in Fusarium oxysporum f.sp. cubense tropical race 4 contributes to virulence towards Cavendish banana
Source: PLoS One. 2018 Oct 22;13(10):e0205896. doi: 10.1371/journal.pone.0205896 (PMC6197647; doi:10.1371/journal.pone.0205896)

Figure S1.

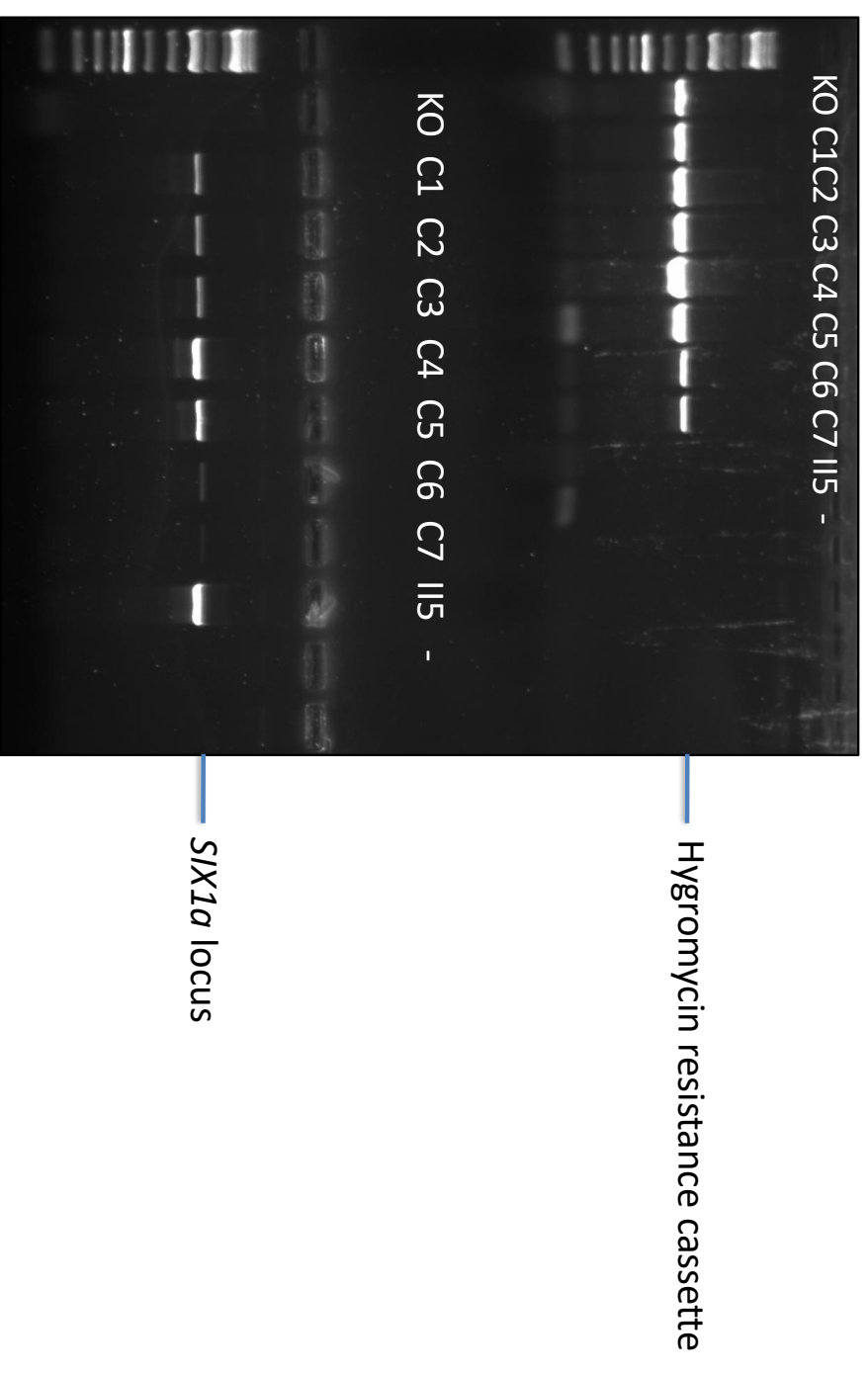

Supplement: S1 Fig — In the FocubΔSIX1a strain (KO), the SIX1a ORF has been replaced by a hygromycin resistance cassette. The FocubΔSIX1a::SIX1a strains (C1-7) have regained the gene by transformation. The upper panel shows the presence of the hygromycin resistance cassette both in the knock-out mutant and in the ectopically transformed strains; the lower panel shows that FocubΔSIX1a has lost the SIX1a locus while in the complemented strains it is present. II5 is the wild type strain. (PDF) [file pone.0205896.s001.pdf]
