# Supplementary material for: A SIX1 homolog in Fusarium oxysporum f.sp. cubense tropical race 4 contributes to virulence towards Cavendish banana
Source: PLoS One. 2018 Oct 22;13(10):e0205896. doi: 10.1371/journal.pone.0205896 (PMC6197647; doi:10.1371/journal.pone.0205896)
Supplement: S2 Fig — (PDF) [file pone.0205896.s002.pdf]

Figure S2

Disease Scoring System

Score	Internal symptom	External symptom	Description	Corm	Leaves
0	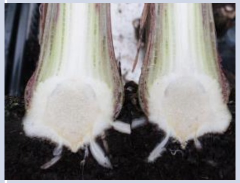	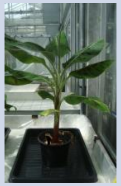	**Healthy**	No browning	Green
1	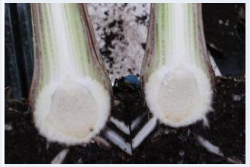	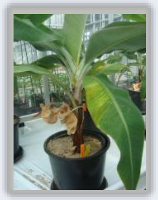	**Chlorosis**	Browning in the root and /or a spot in the corm's edge (1% - 20% browning area)	Mostly green, one leaf start yellowing
2	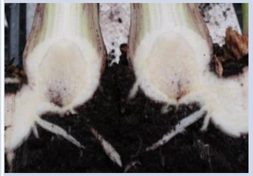	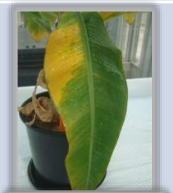	**Yellowing**	Browning from the edge and small blob formation (>20% -40% browning area)	Some leaves have yellowing stripes
3	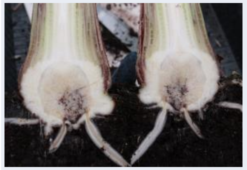	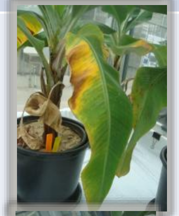	**Necrosis**	Browning in the corm (> 40%-60% browning area)	Yellowing in all leaf surface, or leaf with some necrosis
4	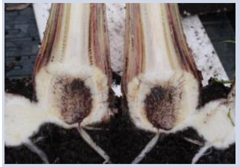	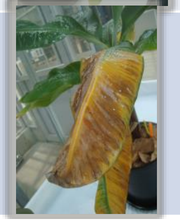	**Wilting**	Browning in the corm (>60%-100% browning area)	Wilting and/or necrosis
