# Supplementary material for: A SIX1 homolog in Fusarium oxysporum f.sp. cubense tropical race 4 contributes to virulence towards Cavendish banana
Source: PLoS One. 2018 Oct 22;13(10):e0205896. doi: 10.1371/journal.pone.0205896 (PMC6197647; doi:10.1371/journal.pone.0205896)

Figure S3

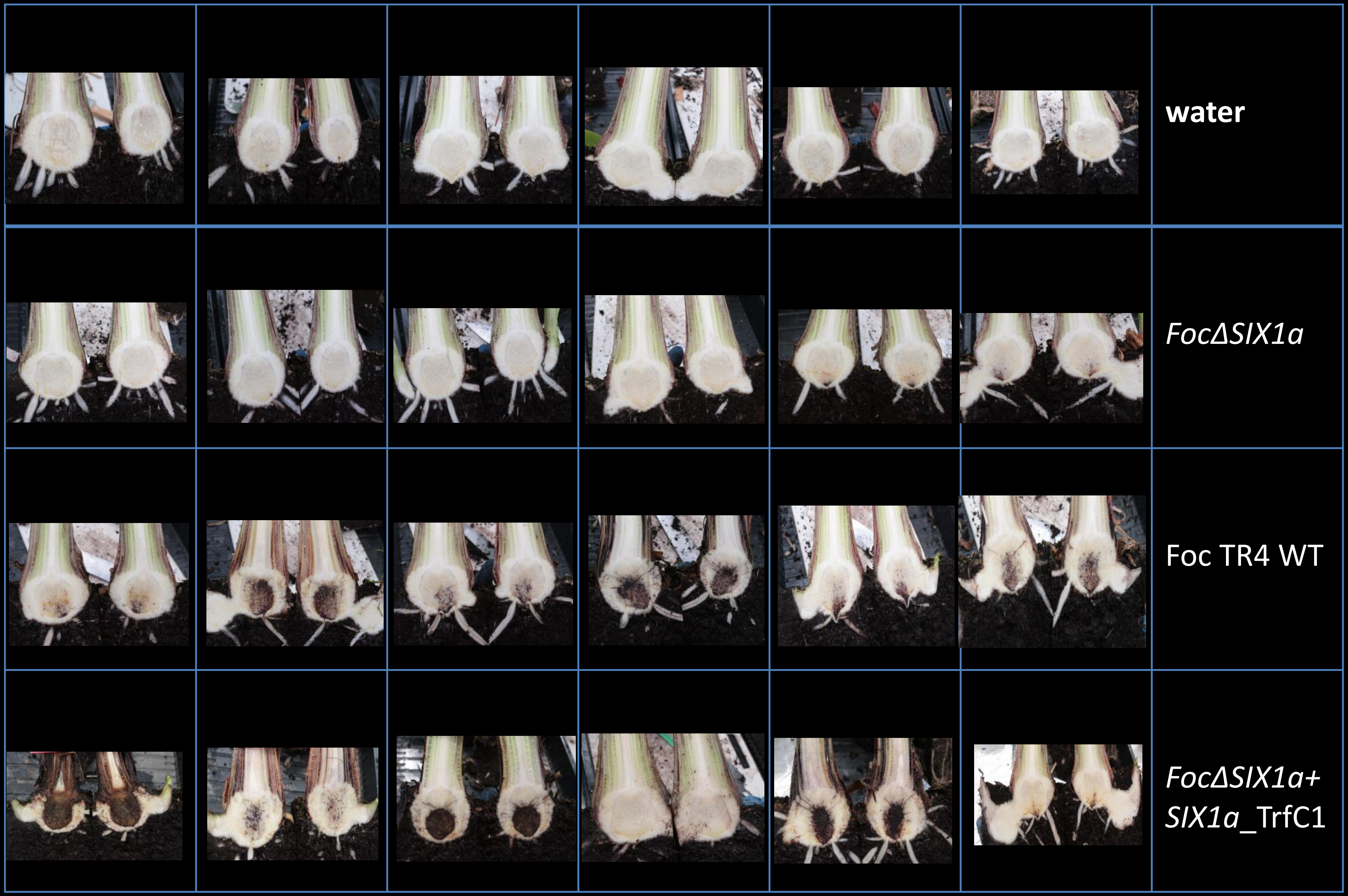

Figure S3 (continued)

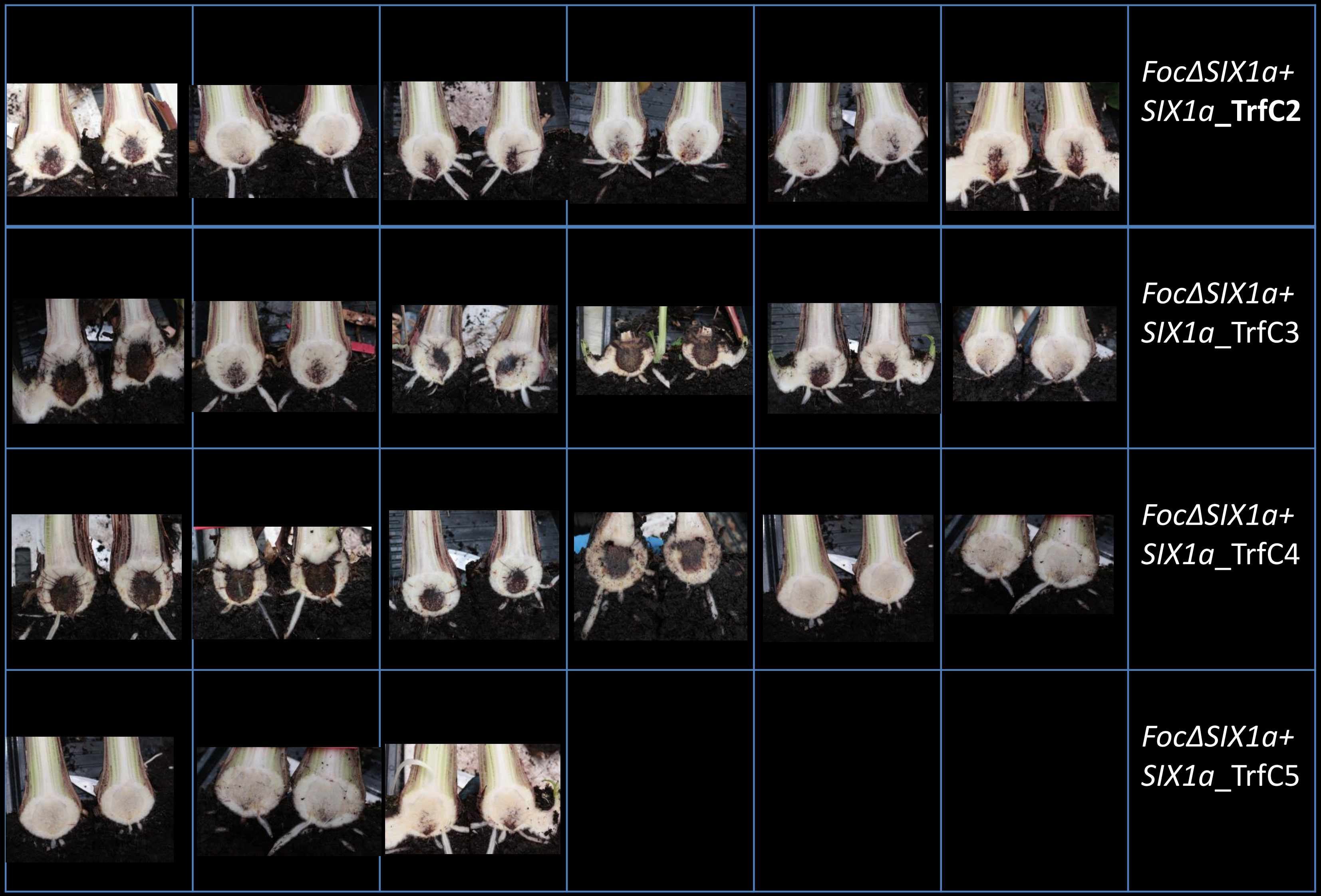

Supplement: S3 Fig — (PDF) [file pone.0205896.s003.pdf]
