# Supplementary material for: A SIX1 homolog in Fusarium oxysporum f.sp. cubense tropical race 4 contributes to virulence towards Cavendish banana
Source: PLoS One. 2018 Oct 22;13(10):e0205896. doi: 10.1371/journal.pone.0205896 (PMC6197647; doi:10.1371/journal.pone.0205896)
Supplement: S1 Table — (DOCX) [file pone.0205896.s004.docx]

Table S1. List of primers being used in this study.

Red colour indicates the restriction site located in the primer sequence.

| **FP#** | **Primers name** | **Primer sequence 5'--->3'** | **Target** |
| --- | --- | --- | --- |
| 5472 | F_UP_FocSIX1b | 5'-ATCCCTCCAGGTTTCAAGCC-3' | Focub-*SIX1b* ORF |
| 5473 | R_DO_FocSIX1b | 5'-GTTTGGAGGGCTTTGGATCG-3' | Focub*-SIX1b* ORF |
| 5474 | F_UP_FocSIX1a | 5'-CCGGATTTTGAGCTTTCGAC3' | Focub*-SIX1a* ORF |
| 5475 | R_DO_FocSIX1a | 5'-GTAGTGTTTGGAGGGCTTTAG-3' | Focub*-SIX1a* ORF |
| 5476 | F_UP_FocSIX1c | 5'-CGGATTTTGAGCTTTCGACAAG-3' | Focub*-SIX1c* ORF |
| 5477 | R_DO_FocSIX1c | 5'-GGAGGGCTTTAGAGCGCAAC-3' | Focub-*SIX1c* ORF |
| 3906 | SMS6 (Hyg-R)(210) | 5'-GCCGATGCAAAGTGCCGATAAACA-3' | Hygromycin resistance cassette |
| 3907 | SMS7 (HYG-F)(850) | 5'-AGAGCTTGGTTGACGGCAATTTCG-3' | Hygromycin resistance cassette |
| 6317 | up_attB3.Foc53_F | 5'-GTGGAGCAATGCCGACCACAG-3' | *SIX1a* upstream attB3 |
| 6318 | do_attB2.FocII5_R | 5'-GTATATTTCAACAGGCAAACAAG-3' | *SIX1a* downstream attB2 |
|  | SIX1attB3 | 5'-GGGGACAACTTTGTATAATAAAGT  TGTCAGGTGACCGGGCCACGTACTG-3' | *SIX1a* promoter |
|  | SIX1attB4 | 5'-GGGACAACTTTGTATAGAAAAGTT  GTTGGAGGGATAATAAGGGGATGTGTC-3' | *SIX1a* promoter |
|  | SIX1attB2 | 5'-GGGGACAGCTTTCTTGTACAAAG  TGGAAGTAGCCAACGGCCTAACAAAGG-3' | *SIX1a* downstream |
|  | SIX1attB1 | 5'-GGGGACTGCTTTTTTGTACAAACT  TGTTCGGGTATATTCACCCATGTTGG-3' | *SIX1a* downstream |
| 6522 | LB_pRW1p+PacI_  FocSIX1a_F | 5'-aaattaattaaCTTGTTGTTGAATTGCGCGG-3' | *SIX1a* 1kb upstream flank |
| 6523 | LB_pRW1p+EcoRI_  FocSIX1a_R | 5'-tttgaattcCTTTCCCTGCAGTTTTCACC-3' | *SIX1a* ~0.8 kb downstream flank |
| 3655 | Ble_R2F | 5'-ATGACCGAGATCGGCGAGCA-3' | Phleomycin resistance cassette |
| 3656 | Ble_R2R | 5'-ACATGCAATTATCTTTGCGAACCC-3' | Phleomycin resistance cassette |
| 6184 | FocSIx1aR-compl-Pac1 | 5'-tttTTAATTAAGTCCAACAGCAG  CTCGAGTAG-3' | *SIX1a* locus |
| 6286 | FocSIX1a-EcoR1-F | 5'-aaagaattcggattttgagctttcgacaag-3' | *SIX1a* locus |
